# A Rational Design of a CoS_2_-CoSe_2_ Heterostructure for the Catalytic Conversion of Polysulfides in Lithium-Sulfur Batteries

**DOI:** 10.3390/ma16113992

**Published:** 2023-05-26

**Authors:** Bin Zhang, Jiping Ma, Manman Cui, Yang Zhao, Shizhong Wei

**Affiliations:** School of Materials Science and Engineering, Henan University of Science and Technology, Luoyang 471003, China; zhangbin15@tsinghua.org.cn (B.Z.); 210321020201@stu.haust.edu.cn (J.M.); c18238073844@163.com (M.C.)

**Keywords:** lithium-sulfur batteries, heterostructure design, lithium polysulfides, catalytic conversion

## Abstract

Lithium-sulfur batteries are anticipated to be the next generation of energy storage devices because of their high theoretical specific capacity. However, the polysulfide shuttle effect of lithium-sulfur batteries restricts their commercial application. The fundamental reason for this is the sluggish reaction kinetics between polysulfide and lithium sulfide, which causes soluble polysulfide to dissolve into the electrolyte, leading to a shuttle effect and a difficult conversion reaction. Catalytic conversion is considered to be a promising strategy to alleviate the shuttle effect. In this paper, a CoS_2_-CoSe_2_ heterostructure with high conductivity and catalytic performance was prepared by in situ sulfurization of CoSe_2_ nanoribbon. By optimizing the coordination environment and electronic structure of Co, a highly efficient CoS_2_-CoSe_2_ catalyst was obtained, to promote the conversion of lithium polysulfides to lithium sulfide. By using the modified separator with CoS_2_-CoSe_2_ and graphene, the battery exhibited excellent rate and cycle performance. The capacity remained at 721 mAh g^−1^ after 350 cycles, at a current density of 0.5 C. This work provides an effective strategy to enhance the catalytic performance of two-dimensional transition-metal selenides by heterostructure engineering.

## 1. Introduction

Lithium-sulfur (Li-S) batteries are expected to be the next generation of storage devices, due to their ultra-high theoretical specific capacity (1673 mAh g^−1^) [1,2,3]. However, liquid intermediate product lithium polysulfides (Li_2_S_x_, 4 ≤ x ≤ 8) are easily dissolved in the electrolyte, leading to a serious shuttle effect, thereby leading to low sulfur utilization, poor cycling stability, and potential safety problems in Li-S batteries [4,5,6]. The insulting nature of sulfur and lithium sulfide (Li_2_S_2_/Li_2_S) results in the sluggish reaction kinetics between Li_2_S_x_ and Li_2_S_2_/Li_2_S, which further accelerate the shuttle effect. In recent years, in order to inhibit the shuttle effect of Li_2_S_x_, researchers have made many improvements to sulfur anodes and separators, mainly through physical confinement [7,8,9,10], chemical adsorption of Li_2_S_x_ [11,12,13,14,15,16] and catalytic conversion of Li_2_S_x_ to Li_2_S_2_/Li_2_S [17,18,19,20,21,22,23]. Although porous carbon materials, such as carbon nanotubes [24,25], porous carbon spheres [26,27,28], graphene [9,11,27,29], and so on, play a certain role in limiting the shuttle effect of Li_2_S_x_, non-polar carbon materials cannot effectively adsorb polar Li_2_S_x_.

Duan’s group analyzed the activation energy for sulfur reduction reactions (SRRs) and revealed the difficulty of different reaction steps [30]. The results show that the activation energy in the initial stage of a SRR is relatively low, indicating that the conversion reaction between solid S_8_ and liquid Li_2_S_x_ is easier. However, the activation energy for the reduction reaction of Li_2_S_x_ to Li_2_S_2_/Li_2_S is large, which means the transformation of liquid Li_2_S_x_ to solid Li_2_S_2_/Li_2_S is difficult. Therefore, the conversion of liquid Li_2_S_x_ to solid Li_2_S is the critical step of a SRR. They demonstrated that an electrocatalyst can effectively the decrease the energy barrier of liquid-solid conversion reactions, and accelerate reduction reactions, thereby reducing accumulation and dissolution of Li_2_S_x_ [30]. Studies have shown that catalytic materials (i.e., metal oxide, metal sulfide, metal nitride, and their heterostructure, etc) increase the reaction kinetics and alleviate the shuttle effect [9,10,11,12,13,14,15,16,17]. 

Compared with other transition-metal compounds, Co-based catalysts, such as CoS_2_ [17], Co_9_S_8_ [31], CoSe_2_ [32,33], Co_3_Se_4_ [34], and CoP [35], can effectively increase the reaction kinetics of an SRR, because of the substantial catalytic ability of Co sites. Moreover, Co-based transition metal compounds are able to bind Li_2_S_x_ via both polar–polar (e.g., Li-S, Li-Se) interaction and Lewis acid-base bonding (Co-S) [17,32], which further increases the catalytic performance; therefore, a Co-based catalyst is one of most promising candidates for Li-S batteries. Generally speaking, the electronic structure and coordination environment of Co sites are widely regarded as the important factors in determining catalytic performance [36]. Compared with single component, two-component heterostructures play an important role in optimizing the electronic structure of the catalyst. Until now, various two-component heterostructures, including VO_2_-VN [37], TiO_2_-MXene [38], and WO_3_-WS_2_ [18], have been developed to enhance the electrochemical performance of Li-S batteries. CoS_2_-CoSe_2_ has been researched in electrocatalytic hydrogen evolution and dye-sensitized solar cells [39,40], however, its catalytic performance in Li-S batteries is not clearly understood. Moreover, the rational design and preparation of the CoS_2_-CoSe_2_ heterostructure, with an optimized electronic structure and coordination environment of Co sites, are still challenging to perform. Herein, we prepared a highly conductive and catalytic CoS_2_-CoSe_2_ heterostructure nanoribbon (NB), via the solvothermal method and in situ sulfurization. During the in situ sulfurization process, some of the Se atoms in CoSe_2_ are replaced by S atoms. The strong binding between CoS_2_ and CoSe_2_ promotes the rapid electron transfer and optimizes the electronic structure of Co sites. Moreover, CoSe_2_ converts from a stable cubic phase to a metastable orthorhombic phase after in situ sulfurization, which changes the coordination environment of Co sites, thereby increasing the catalytic performance. By using added CoS_2_-CoSe_2_ and graphenes to modify separator, the battery exhibits excellent rate and cycle performance. The Li-S battery delivers a good capacity of 612 mAh g^−1^ after 400 cycles at 1 °C. 

## 2. Materials and Methods

Synthesis of CoSe_2_ NBs. CoSe_2_ NBs were prepared by the solvothermal method [41]. The specific steps are as follows. (1) First, 1 mmol of cobalt acetate (Co(AC)_2_H_2_O, 0.249 g) was added to 5 mL of deionized water and stirred until Co(AC)_2_H_2_O was completely dissolved, followed by adding 8 mL of graphene oxide dispersion (0.3 mg mL^−1^). A small amount of graphene oxide was added to prevent agglomeration of CoSe_2_ NBs. The dispersion solution was treated by strong ultrasonic for 2 h. (2) Then, 26 mL diethylenetriamine and 1 mmol sodium selenite (Na_2_SeO_3_, 0.173 g) were added to the dispersion solution and stirred for 3 h until Na_2_SeO_3_ was completely dissolved. (3) The completely dissolved solution was transferred to a 50 mL reactor and reacted at 180 °C for 16 h, after which the product was cleaned with deionized water. Finally, black powder was obtained by freeze drying. The as-prepared sample was labeled as CoSe_2_.

Synthesis of CoS_2_-CoSe_2_ heterostructure. The detailed steps are as follows. (1) 30 mg CoSe_2_ and 150 mg thiourea (CH_4_N_2_S) were placed in two separate boats of tubular furnace. CoSe_2_ was placed in central zone and CH_4_N_2_S was placed in the upstream of flow. (2) The heat treatment was performed at 350 °C for 0.5 h under an argon atmosphere. The as-prepared sample was labeled as CoS_2_-CoSe_2_. Other CoS_2_-CoSe_2_ heterostructures were synthesized using different contents of thiourea, and they were labeled as CoS_2_-CoSe_2_-1 (thiourea: 50 mg), CoS_2_-CoSe_2_-2 (thiourea: 100 mg), and CoS_2_-CoSe_2_-3 (thiourea: 200 mg).

Synthesis of cathode and modified separators. Synthesis of CNT/S cathode: The sulfur/carbon composite was prepared by heat treatment at 155 °C for 12 h after mixing the nano-sulfur powder and carbon nanotubes at a mass ratio of 7.5:2.5. Then, the sulfur/carbon composite, Super P, and PVDF were mixed at a mass ratio of 8:1:1, and NMP solvent was added and stirred for 6 h. The stirred slurry was coated on aluminum foil, dried at 60 °C for 12 h and cut into a disk with a diameter of 12 mm. The loading mass of sulfur was 1 mg cm^−2^. Synthesis of modified separators: Firstly, a dispersion liquid containing 3 mg of CoS_2_-CoSe_2_ powder, 14 mg graphene (GN), and 100 mL of ethanol was obtained via strong ultrasonic for 2 h. The dispersion was vacuum filtered on the blank separator and dried naturally for 12 h. Then, the separator loaded with CoS_2_-CoSe_2_/GN and was cut into discs with a diameter of 19 mm, and the mass loading of CoS_2_-CoSe_2_/GN on the disc was about 0.17 mg cm^−2^. The CoSe_2_/GN composite separator was prepared by the same method. 

Battery assembly and electrochemical measurements. This was performed using CNT/S as the cathode, a catalyst/GN modified separator, lithium foil as the anode, and 1 M LiTFSI dissolved in a DME/DOL (volume ratio is 1:1) with 2% LiNO_3_ (mass ratio) as the electrolyte to assemble Li-S coin cells. The electrolyte/sufur (E/S) ratio is was 15 µL mg^−2^. Galvanostatic charge–discharge curves were measured with battery test system (Land CT2001A). CV profiles were performed on VMP3 electrochemical workstation.

Materials Characterization. The morphologies of the samples were examined by scanning electron microscopy (SEM SU8010) and transmission electron microscopy (FEI Tecnai G2 F30). X-ray diffraction (XRD) patterns of the samples were carried out on a Bruker D8 Advance diffractometer using Cu Ka radiation. The N_2_ adsorption–desorption isotherm of the samples was measured using a Belsorp Max II analyzer. X-ray photoelectron spectroscopy (XPS) analyses were carried on a PHI 5000 VersaProbe II spectrometer using monochromatic Al K(alpha) X-ray source.

## 3. Results and Discussion

**Figure 1:**
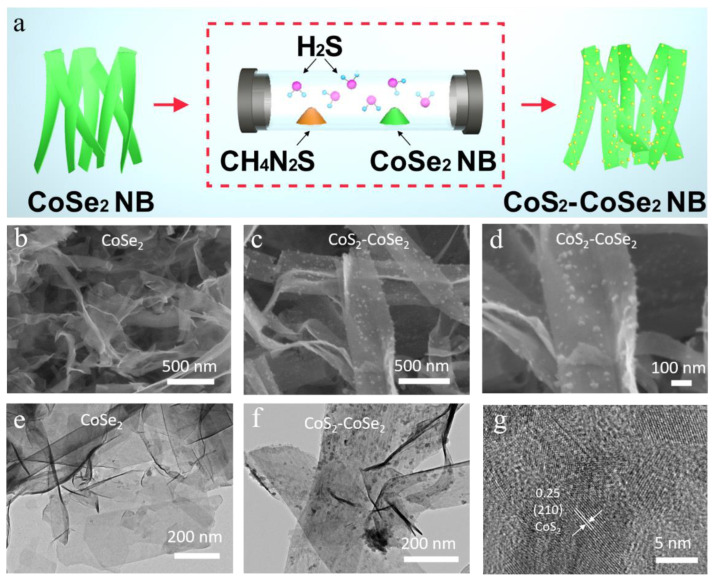
(**a**) Schematic diagram of the synthesis process of CoS_2_-CoSe_2_; SEM images of (**b**) CoSe_2_ and (**c**) CoS_2_-CoSe_2_; (**d**) SEM image of CoS_2_-CoSe_2_; TEM images of (**e**) CoSe_2_ and (**f**) CoS_2_-CoSe_2_; (**g**) HR-TEM image of CoS_2_-CoSe_2_.

The CoSe_2_ NBs were prepared by solvothermal method; some protonated ammonia molecules were intercalated within the CoSe_2_ NB [41]. The in situ sulfurization of CoSe_2_ into CoS_2_-CoSe_2_ heterostructure can be easily realized by heat treatment. In a typical preparation, thiourea and CoSe_2_ NBs were first placed in two separate crucibles with different mass ratios, followed by heat treatment. Then, the H_2_S vapor was formed to react with CoSe_2_ to realize the in situ sulfurization (Figure 1a). Note that the content of the CoS_2_ was related to the thiourea content and gradually increased with higher thiourea contents (Figure S1). More synthesis details can be found in the Experimental section. The morphologies of CoSe_2_ and CoS_2_-CoSe_2_ were observed by scanning electron microscopy (SEM) and transmission electron microscopy (TEM), as shown in Figure 1b,e, The two-dimensional nanoribbon is flexible and transparent, indicating that ultra-thin nanoribbons exhibit good electrical conductivity. The nanoribbon was then sulfurized in situ through heat treatment using thiourea as sulfur source. As shown in Figure 1c,d, the smooth nanoribbon surface becomes rough after sulfurization, and a large number of nanoparticles were grown in situ on the nanoribbon of CoSe_2_. The nanoparticles were uniformly dispersed on the surface of the nanoribbon and were less than 20 nm in size (Figure 1f). The uniform dispersion of nanoparticles may be ascribed to the following two reasons. Firstly, uniform selenium vacancies were formed on the surface of CoSe_2_ during heat treatment, and the selenium vacancies were rapidly replaced by sulfur to generate CoS_2_ nanoparticles. Secondly, the surface of CoSe_2_, prepared by the solvothermal method, contained a large number of amino functional groups, which could act as nucleation site, to promote the uniform growth of CoS_2_ on the surface of CoSe_2_. The nanoparticles were still firmly anchored on the CoSe_2_ nanoribbon after strong ultrasonic dispersion during the preparation process of the TEM sample, indicating that the strong bonding force existed between the CoS_2_ and CoSe_2_, which was due to the fact that CoS_2_ nanoparticles were attached in situ on selenium vacancies or amino functional groups. As shown in Figure 1g, high-resolution TEM confirms that the nanoparticles on the nanoribbon were CoS_2_, and a lattice spacing of 0.25 nm corresponded to the (210) plane of CoS_2_.

**Figure 2:**
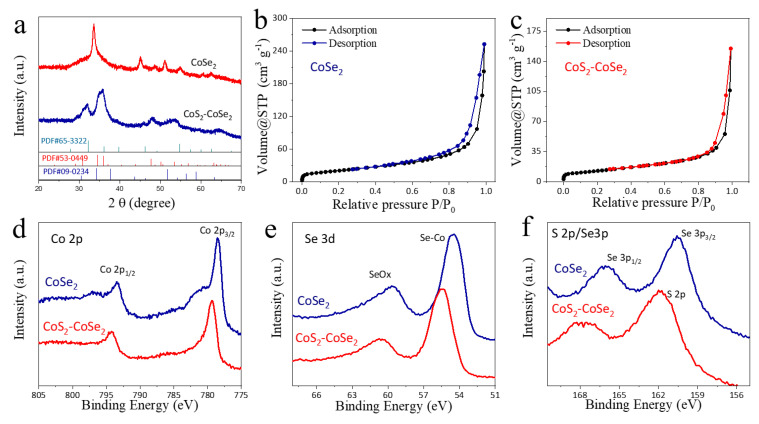
XRD patterns of (**a**) CoSe_2_ and CoS_2_-CoSe_2_; nitrogen adsorption and desorption curves of (**b**) CoSe_2_ and (**c**) CoS_2_-CoSe_2_; XPS spectra of (**d**) Co 2p; (**e**) Se 3d; and (**f**) S 2p of CoSe_2_ and CoS_2_-CoSe_2_.

X-ray diffraction (XRD) was used to study the phases of CoSe_2_ and CoS_2_-CoSe_2_. As shown in Figure 2a, the CoSe_2_ nanoribbons prepared by solvothermal reaction are cubic phase (JCPDS No.09-0234). The characteristic peak of CoS_2_ appears at 32.3° (JCPDS No.65-3322), after in situ sulfurization, which further confirms the formation of CoS_2_-CoSe_2_ [39]. Meanwhile, the stable cubic CoSe_2_ was transformed into the metastable orthorhombic CoSe_2_ (JCPDS No.53-0449). The formation of orthorhombic CoSe_2_ was accompanied by the rotation of the Se-Se bond, which alters the coordination environment of Co sites. This metastable orthorhombic CoSe_2_ showed higher electrocatalytic activity than the stable cubic phase [42]. Nitrogen adsorption desorption tests were conducted on CoSe_2_ and CoS_2_-CoSe_2_, and the results are shown in Figure 2b,c. There was no difference in adsorption–desorption curve types between CoSe_2_ and CoS_2_-CoSe_2_, and both of them had a small number of mesoporous pores. According to a Brunauer–Emmett–Teller theoretical calculation, the specific surface areas of CoSe_2_ and CoS_2_-CoSe_2_ are 74 and 45 m^2^ g^−1^, respectively. The formation of the CoS_2_-CoSe_2_ heterostructure was accompanied by the decrease of a specific surface area, which was caused by the growth of granular CoS_2_ on the surface of ultra-thin nanoribbons.

The surface chemistries of CoS_2_-CoSe_2_ and CoSe_2_ were tested and compared by X-ray photoelectron spectroscopy. Figure 2d shows the Co 2p spectra of CoS_2_-CoSe_2_ and CoSe_2_, in which two peaks at 778.46 and 793.36 eV are attributed to Co 2p_3/2_ and Co 2p_1/2_ of CoSe_2_, after the sulfurization process, and the two Co-Se bonds move to higher binding energy positions, which are 779.19 and 794.09 eV, respectively. During the in situ sulfurization process, some Se atoms in CoSe_2_ are replaced with S atoms, which is accompanied by the formation of a CoS_2_-CoSe_2_ heterostructure. The Co 2p_3/2_ spectrum moves to the higher binding energy position with 0.73 eV, indicating a Co element under oxidized change. Moreover, electron transfer occurs between the heterostructure, and a strong interaction exists between CoS_2_ and CoSe_2_. The analysis of the Se 3d fine spectra of the two materials shows that 54.7 and 59.6 eV correspond to Se_2_^2−^ and SeO_x_, respectively; after the conversion of CoSe_2_ to CoS_2_-CoSe_2_, Se 3d moves to higher binding energy position (Figure 2e). The fine spectrum of S 2p confirms the existence of CoS_2_ and thiosulfate/sulfate (Figure 2f). Above all, the electronic structure and coordination environment of Co were changed after the in situ sulfurization.

**Figure 3:**
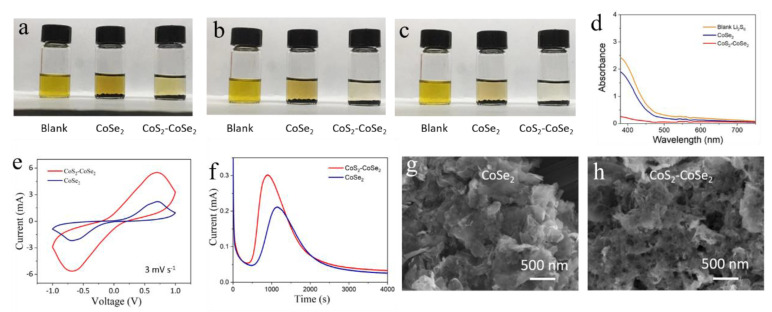
Digital photographs of Li_2_S_6_ adsorption test of CoSe_2_ and CoS_2_-CoSe_2_ at different times: (**a**) 0.5 h; (**b**) 3 h; (**c**) 5 h; (**d**) UV−vis spectra of the Li_2_S_6_ solution after 5 h of adsorption; (**e**) CV curves of CoSe_2_ and CoS_2_-CoSe_2_ symmetrical cells; (**f**) potentiostatic discharge profiles of the cells with CoSe_2_ and CoS_2_-CoSe_2_. Electrode morphology of (**g**) CoSe_2_ and (**h**) at the beginning of deposition.

To investigate the polysulfide adsorption capability of CoS_2_-CoSe_2_, and CoSe_2_ toward Li_2_S_x_, visual static Li_2_S_6_ adsorption tests were performed on CoS_2_-CoSe_2_ and CoSe_2_. As shown in Figure 3a–c, the yellow Li_2_S_6_ solution of CoS_2_-CoSe_2_ begins to fade after 0.5 h and becomes clear after 3 h; however, the color of CoSe_2_ began to fade after 5 h, proving that the CoS_2_-CoSe_2_ heterostructure has a strong adsorption ability with Li_2_S_6_. The UV–vis technique was used to investigate the polysulfide adsorption capability of different materials. The peak of the UV–vis spectrum in the solution with CoS_2_-CoSe_2_ exhibited the weakest intensity, indicating Li_2_S_6_ immobilization of CoS_2_-CoSe_2_ (Figure 3d). This was because the electronic structure of Co in the CoS_2_-CoSe_2_ heterostructure was optimized and the Co coordination environment of metal was changed, increasing the adsorption capability of Li_2_S_6_. Furthermore, the formation of CoS_2_ also increased the adsorption ability. 

The catalytic activity of the materials was evaluated through cyclic voltammogram (CV) curves of symmetrical cells, using an Li_2_S_6_ electrode. As shown in Figure 3e, compared with CoSe_2_, the CoS_2_-CoSe_2_ symmetrical cell has a higher current density, which indicates that CoS_2_-CoSe_2_ accelerates the catalytic conversion of Li_2_S_x_ and improves the utilization of sulfur. Moreover, the Li_2_S deposition experiment was conducted for CoS_2_-CoSe_2_ and CoSe_2_, to illustrate the catalytic activity. Catalysts were loaded on carbon fiber paper, and an Li_2_S_8_ solution was added on the catalysts. The coin cell was assembled using carbon fiber paper loaded with a catalyst, Li_2_S_8_ as the cathode, and lithium foil as the anode. The coin cell was galvanostatically discharged to 2.07 V and then potentiostatically discharged at 2.06 V. The potentiostatic discharge curves of cells with CoSe_2_ and CoS_2_-CoSe_2_ are shown in Figure 3f. The peak currents of CoSe_2_ and CoS_2_-CoSe_2_ were 0.21 and 0.3 mA, respectively. Moreover, compared with CoSe_2_, the deposition time of Li_2_S_x_ for CoS_2_-CoSe_2_ was shorter, demonstrating that the CoS_2_-CoSe_2_ delivered better catalytic performance toward Li_2_S_x_. The morphology of the electrode was observed at the same deposition time point, as shown in Figure 3g,h. No obvious Li_2_S was present on the surface of CoSe_2_; in comparation, a large area of Li_2_S had already emerged on the surface of CoS_2_-CoSe_2_, confirming that the CoS_2_-CoSe_2_ heterostructure improved the conversion efficiency of Li_2_S_x_ to Li_2_S_2_/Li_2_S. The morphologies of the CoSe_2_ and CoS_2_-CoSe_2_ electrodes after Li_2_S deposition were also investigated. As shown in Figure S2, a large amount of Li_2_S could be observed on the surface of the CoS_2_-CoSe_2_ electrode, while only a few deposits were detected on the on the surface of the CoSe_2_ electrode, further confirming that CoS_2_-CoSe_2_ was beneficial for the deposition of Li_2_S.

Above all, compared with CoSe_2_, the adsorption and catalytic conversion ability of CoS_2_-CoSe_2_ have been largely improved, due to the presence of CoS_2_, which may be attributed to two reasons. Firstly, the heterostructure activated the Co catalytic activity on the surface of the CoSe_2_, and the formed hetero-interface ensured the rapid electron transfer of and Li-ion diffusion between CoS_2_ and CoSe_2_. Secondly, the orthorhombic CoSe_2_ changed the coordination environment of the Co, increasing the adsorption sites and catalytic activity. 

**Figure 4:**
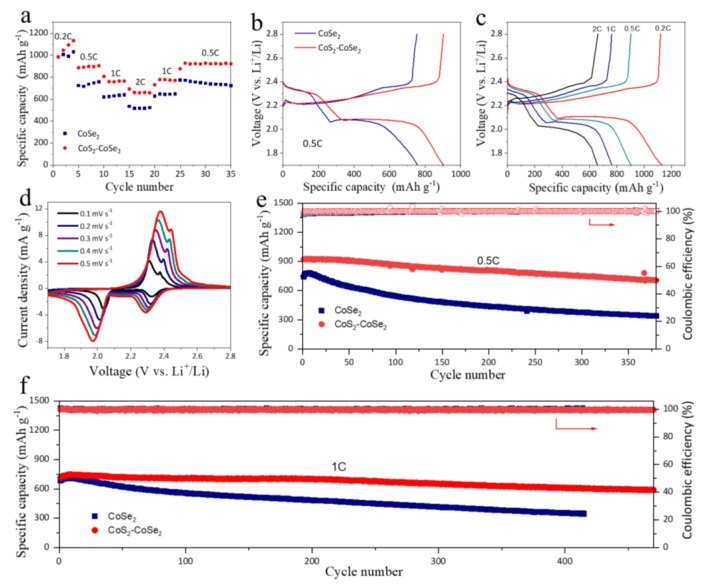
(**a**) Rate performance of different batteries at different current densities from 0.2 to 2 C. (**b**) Comparison of charge–discharge curves of batteries using different separators at a current density of 0.5 C. (**c**) Charge–discharge curves of battery using CoS_2_-CoSe_2_/GN separator at different current densities. (**d**) CV curves of cell using CoS_2_-CoSe_2_/GN separator at different scan speeds. The cycling performance of batteries using CoS_2_-CoSe_2_/GN and CoSe_2_/GN separators at (**e**) 0.5 C and (**f**) 1 C, the arrows represent the coulombic efficiency.

To investigate the electrochemical performance of Li-S batteries with CoSe_2_ and CoS_2_-CoSe_2_, the CoS_2_-CoSe_2_/GN or CoSe_2_/GN modified separator was applied to the Li-S batteries. The modified separator had a loading of 0.17 mg cm^−2^ and a thickness of ~11 μm (Figure S3). The rate performance of Li-S batteries with CoS_2_-CoSe_2_ and CoSe_2_ were tested under different current densities. As shown in Figure 4a, the battery with CoS_2_-CoSe_2_ had discharge capacities of 1133, 905, 765, and 658 mAh g^−1^ at 0.2, 0.5, 1, and 2 C current densities, respectively. The discharge capacities of the battery with the CoSe_2_ cell are 1031, 757, 639, and 524 mAh g^−1^, indicating that the CoS_2_-CoSe_2_ improved the sulfur utilization. Figure 4b displays the charge–discharge curves of the two batteries. When the current density was 0.5 °C, the battery with CoS_2_-CoSe_2_ had a longer discharge platform, which proves that CoS_2_-CoSe_2_ promotes the conversion of Li_2_S_x_ and improves the utilization of sulfur. By analyzing the charging curves of the two batteries, it can be seen that the battery with CoS_2_-CoSe_2_ can reduce the energy barrier of conversion from Li_2_S_2_/Li_2_S to Li_2_S_x_, indicating that CoS_2_-CoSe_2_ can promote its conversion reaction. Figure 4c shows the charge–discharge curves of the battery using CoS_2_-CoSe_2_ at different current densities. The discharge curves at different current densities contained two discharge platforms, corresponding to the reduction reaction from solid S_8_ to liquid Li_2_S_x_, and Li_2_S_x_ to Li_2_S_2_/Li_2_S. CV tests were conducted at different scanning speeds for the battery with CoS_2_-CoSe_2_. It can be seen that, even at the scanning speed of 0.5 mV s^−1^, there were still two obvious reduction peaks and oxidation peaks, indicating that the battery had excellent rate performance (Figure 4d).

Figure 4e shows the long cycle performance of the two batteries at a current density of 0.5 C. The battery using the CoS_2_-CoSe_2_ had an initial capacity of 921 mAh g^−1^ and a capacity of 722 mAh g^−1^ after 350 cycles, corresponding to a capacity decay rate of 0.062% per cycle. The cell using the CoSe_2_ had an initial capacity of 740 mAh g^−1^ and a capacity of 352 mAh g^−1^ after 350 cycles, corresponding to a capacity decay rate of 0.15% per cycle. Although CoSe_2_ has a high specific surface area, its poor Li_2_S_x_ adsorption and catalytic ability resulted in poor cycling stability. CoS_2_-CoSe_2_ has good adsorption and catalytic conversion ability toward Li_2_S_x_, which improved the capacity and cycle stability of the battery. Figure 4f shows the long cycle performance of the two batteries at 1 C. After 400 cycles, the battery with CoS_2_-CoSe_2_ had a capacity of 612 mAh g^−1^, while the cell with CoSe_2_ only had a capacity of 353 mAh g^−1^, which demonstrates that CoS_2_-CoSe_2_ can improve the utilization of sulfur and alleviate the shuttle effect of Li_2_S_x_. We also compared the cycle performance of batteries with other Co-based composite electrodes, measured at 0.5/1 C (Table S1), indicating that the battery with CoS_2_-CoSe_2_ had a longer cycling life.

## 4. Conclusions

In conclusion, we prepared CoSe_2_ nanoribbons with a high specific surface area and high conductivity, by the solvothermal method, and then prepared a CoS_2_-CoSe_2_ heterostructure by in situ sulfurization. The strong interaction between the two components ensures rapid electron transfer and Li-ion diffusion. The formation of a CoS_2_-CoSe_2_ heterostructure optimizes the electronic structure of Co, and simultaneously converts its phase from a stable cubic phase into the metastable orthorhombic phase, which accompanies the change of the coordination environment of Co. As a result, CoS_2_-CoSe_2_ significantly increases adsorption and catalytic ability, thereby improving the electrochemical performance of the Li-S battery. This work provides an easy way to construct highly efficient heterostructure catalysts for Li-S batteries by the in situ sulfurization of a metal sulfide heterostructure. 

## Data Availability

All relevant data are within the manuscript and its Additional files.

## Supplementary Materials

The following supporting information can be downloaded at: https://www.mdpi.com/article/10.3390/ma16113992/s1. References [43,44,45] are cited in the supplementary materials.
