# Supplementary material for: A Rational Design of a CoS2-CoSe2 Heterostructure for the Catalytic Conversion of Polysulfides in Lithium-Sulfur Batteries
Source: Materials (Basel). 2023 May 26;16(11):3992. doi: 10.3390/ma16113992 (PMC10254419; doi:10.3390/ma16113992)
Supplement: Supplementary file 1 [file materials-16-03992-s001.zip › materials-2362876-supplementary.pdf]

# Supporting Information

## Rational Design of $CoS_2$-$CoSe_2$ Heterostructure for Catalytic Conversion of Polysulfides in Lithium-Sulfur Batteries

*Bin Zhang, Jiping Ma, Manman Cui, Yang Zhao[*] and Shizhong Wei[*]*

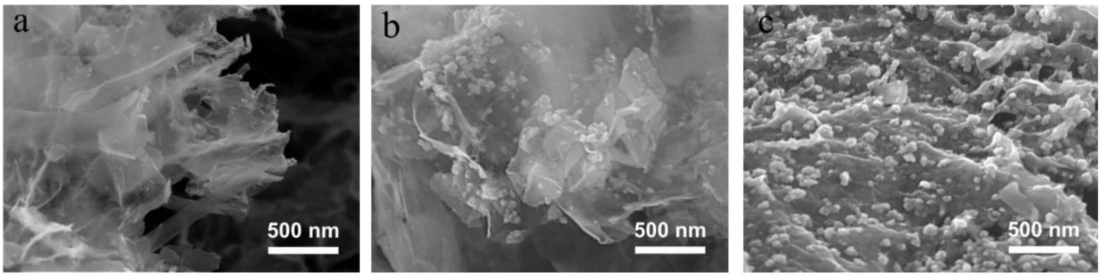


Figure S1. SEM images of $CoS_2$-$CoSe_2$ with different thiourea contents during the sulfurization process: (a) 50 mg, (b) 100 mg, and (c) 200 mg

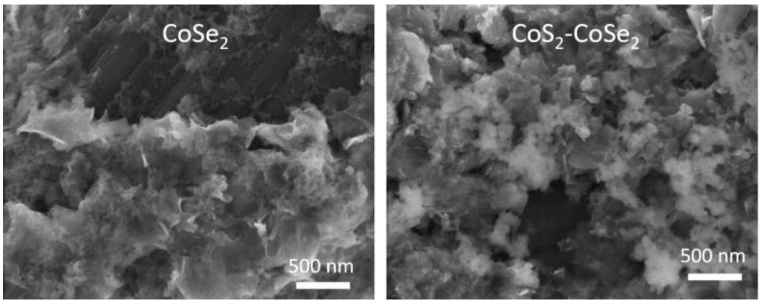


Figure S2. SEM images of $CoSe_2$ and $CoS_2$-$CoSe_2$ electrodes after $Li_2S$ deposition.

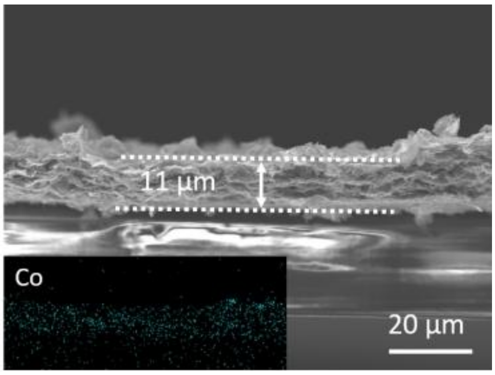


Figure S3. Cross-section image of $CoS_2$-$CoSe_2$/GN composite interlayer

Table S1. Performance comparison of this work with other related works

| Materials | Sulfur loading | Rate | Cycle number | References |
|---|---|---|---|---|
| $CoS_2$ + G | 0.4 mg cm$^{-2}$ | 0.5C | 100 | [1] |
| $CoSe_2$/G | 2.69 mg cm$^{-2}$ | 0.5C | 100 | [2] |
| CS@HPP | | 1C | 300 | [3] |
| N-CN-750@$Co_3Se_4$-0.1 m | | 0.5C | 200 | [4] |
| N-$CoSe_2$ | | 0.2C | 250 | [5] |
| {$Co_4W_{18}$}/rGO | | 1C | 100 | [6] |
| $Ni_{0.1}Zn_{0.1}Co_{0.8}Se_2$ | | 1C | 400 | [7] |
| CoP-CNT | 3 mg cm$^{-2}$ | 1C | 200 | [8] |
| $CoS_2$-$CoSe_2$ | 1 mg cm$^{-2}$ | 1C | 450 | Our Work |

[1] Yuan Z, Peng H-J, Hou T-Z, Huang J-Q, Chen C-M, Wang D-W, Cheng X-B, Wei F, Zhang Q. Powering Lithium-Sulfur Battery Performance by Propelling Polysulfide Redox at Sulfiphilic Hosts. *Nano Lett.* **2016**, *16*, 519-527. https://doi.org/10.1021/acs.nanolett.5b04166.

[2] Yuan H, Peng H-J, Li B-Q, Xie J, Kong L, Zhao M, Chen X, Huang J-Q, Zhang Q. Conductive and Catalytic Triple-Phase Interfaces Enabling Uniform Nucleation in High-Rate Lithium-Sulfur Batteries. *Adv. Energy Mater.* **2019**, *9*, 1802768. https://doi.org/10.1002/aenm.201802768.

[3] Ye Z, Jiang Y, Li L, Wu F, Chen R. A High-Efficiency Cose Electrocatalyst with Hierarchical Porous Polyhedron Nanoarchitecture for Accelerating Polysulfides Conversion in Li-S Batteries. *Adv. Mater.* **2020**, *32*, 2002168. https://doi.org/10.1002/adma.202002168.

[4] Cai D, Liu B, Zhu D, Chen D, Lu M, Cao J, Wang Y, Huang W, Shao Y, Tu H, Han W. Ultrafine $Co_3se_4$ Nanoparticles in Nitrogen-Doped 3D Carbon Matrix for High-Stable and Long-Cycle-Life Lithium Sulfur Batteries. *Adv. Energy Mater.* **2020**, *10*, 1904273. https://doi.org/10.1002/aenm.201904273.

[5] Wang M, Fan L, Sun X, Guan B, Jiang B, Wu X, Tian D, Sun K, Qiu Y, Yin X, Zhang Y, Zhang N. Nitrogen-Doped $CoSe_2$ as a Bifunctional Catalyst for High Areal Capacity and Lean Electrolyte of Li-S Battery. *ACS Energy Lett.* **2020**, *5*, 3041-3050. https://doi.org/10.1021/acsenergylett.0c01564.

[6] Lei J, Fan X-X, Liu T, Xu P, Hou Q, Li K, Yuan R-M, Zheng M-S, Dong Q-F, Chen J-J. Single-Dispersed Polyoxometalate Clusters Embedded on Multilayer Graphene as a Bifunctional Electrocatalyst for Efficient Li-S Batteries. *Nat. Commun.* **2022**, *13*. https://doi.org/10.1038/s41467-021-27866-5.

[7] Chen L, Xu Y, Cao G, Sari HMK, Duan R, Wang J, Xie C, Li W, Li X. Bifunctional Catalytic Effect of $CoSe_2$ for Lithium-Sulfur Batteries: Single Doping Versus Dual Doping. *Adv. Funct. Mater.* **2022**, *32*, 2107838. https://doi.org/10.1002/adfm.202107838.

[8] Zhong Y, Yin L, He P, Liu W, Wu Z, Wang H. Surface Chemistry in Cobalt Phosphide-Stabilized Lithium-Sulfur Batteries. *J. Am. Chem. Soc.* **2018**, *256*, 1455-1459.
